# ROS-Mediated Cell Cycle Arrest and Apoptosis Induced by Zearalenone in Mouse Sertoli Cells via ER Stress and the ATP/AMPK Pathway

**DOI:** 10.3390/toxins10010024

**Published:** 2018-01-01

**Authors:** Wang-Long Zheng, Bing-Jie Wang, Ling Wang, Yu-Ping Shan, Hui Zou, Rui-Long Song, Tao Wang, Jian-Hong Gu, Yan Yuan, Xue-Zhong Liu, Guo-Qiang Zhu, Jian-Fa Bai, Zong-Ping Liu, Jian-Chun Bian

**Affiliations:** 1 College of Veterinary Medicine, Yangzhou University, Yangzhou 225009, China; zhengwanglong@163.com (W.-L.Z.); wangbingjie123188@163.com (B.-J.Y.); wl7590@163.com (L.W.); zouhui@yzu.edu.cn (H.Z.); srlbio@163.com (R.-L.S.); wtao6550@yzu.edu.cn (T.W.); jhgu@yzu.edu.cn (J.-H.G.); yuanyan@yzu.edu.cn (Y.Y.); liuxuezhong68@163.com (X.-Z.L.); yzgqzhu@yzu.edu.cn (G.-Q.Z.); 2 Jiangsu Co-Innovation Center for Prevention and Control of Important Animal Infectious Diseases and Zoonoses, Yangzhou 225009, China; 3 Joint International Research Laboratory of Agriculture and Agri-Product Safety, the Ministry of Education of China, Yangzhou University, Yangzhou 225009, China; 4 Kansas State Veterinary Diagnostic Laboratory, Kansas State University, 1800 Denison Avenue, Manhattan, KS 66506, USA; jbai@vet.k-state.edu; 5 Lianyungang Husbandry and Veterinary Station, Lianyungang 222001, China; lygsyp8@126.com

**Keywords:** Zearalenone, cell cycle, cell apoptosis, ROS, ER stress, AMPK signaling, Sertoli cells, Zearalenone induced the cell cycle arrest and apoptosis through ROS-ER stress-AMPK pathway in mouse sertoli cells.

## Abstract

Zearalenone (ZEA) can perturb the differentiation of cells, reduce the generation of reproductive cells and induce a death of germ cells, but the molecular mechanism remains unclear. In order to investigate the potential mechanism of ZEA-induced cell cycle arrest and apoptosis, we studied the effects of ZEA on cell proliferation, cell-cycle distribution, cell-cycle-related proteins, cell death, cell apoptosis, ROS generation and the ATP/AMPK pathway in Sertoli cells. The role of ROS, ER stress and the ATP/AMPK pathway in ZEA-induced cell-cycle arrest and cell apoptosis was explored by using the antioxidant NAC, ER stress inhibitor 4-PBA and the AMPK inhibitor dorsomorphin, respectively. The results revealed that ZEA inhibited the cell proliferation, influenced the distribution of the cell cycle and induced cell apoptosis through the ATP/AMPK pathway. The ATP/AMPK pathway was regulated by ER stress that was induced by ROS generation after exposure to ZEA. Taking these together, this study provided evidence that ROS regulated the process of ZEA-induced cell cycle arrest and cell apoptosis through ER stress and the ATP/AMPK signal ways.

## 1. Introduction

Zearalenone (ZEA), produced by several Fusarium species, can exert estrogen-like activity and cause reproductive dysfunction due to its chemical structure [1,2]. ZEA can perturb the differentiation of cells, reduce the generation of reproductive cells and induce the death of germ cells [3,4]. Human and animal exposure to ZEA may be attributed to the consumption of cereal grains that have been contaminated by toxigenic molds that may produce mycotoxins and their metabolites. Furthermore, animal-derived food including meat, milk and eggs from animals exposed to ZEA or that have been given α-Zearalanol (α-ZAL) to promote their growth may also contain several mycotoxins and their metabolites [5,6].

Accumulated evidence has suggested that ZEA exerts different mechanisms of toxicity in different dose and cell types. A low dose of ZEA can promote the cell cycle process and increase the cell viability in breast cancer cells [7]. Furthermore, a low dose of ZEA has potential tumor-promoting activity, which can promote cell proliferation and division via down-regulating apoptosis [8,9]. However, several studies have showed that treatment with a high dose of ZEA can activate autophagy and apoptosis in mouse and goat Leydig cells [10,11]. ZEA can induce cell death and apoptosis in RAW 264.7 macrophages and the male reproductive system in mice through the endoplasmic reticulum (ER) stress pathway [4,12]. Causing oxidative stress and producing DNA damage were important methods for ZEA and its metabolites to exert their cytotoxicity in HepG2 cells [13]. ZEA induced cellular proliferation may contribute to strong estrogenic effects [7]. On the contrary, in high concentrations it exerts other mechanisms of toxicity such as oxidative stress and damage [14]. 

Most of the studies on ZEA-induced cell death usually focused on ZEA-induced cell apoptosis and autophagy and there is a lack of the studies exploring the mechanisms of ZEA-inhibited cell proliferation. Thus, the aim of this study was to investigate the mechanisms of ZEA-induced cell cycle arrest and apoptosis. In the current study, the results showed that the upstream control signal of ZEA-induced ER stress was ROS generation, including ROS-mediated cell cycle arrest and apoptosis through ER stress pathway in TM4 cells. Although many studies have showed that ZEA-induced cell apoptosis via ER stress, we found that besides those classical pathways of ER stress-inducing apoptosis, the ATP/AMPK signal way was also mediated by ER stress in the process of ZEA-induced cell cycle arrest and apoptosis.

## 2. Results

### 2.1. ZEA Inhibits the Cell Proliferation and Cause Cell Cycle Arrest at the G2/M Phase in TM4 Cells

**Figure 1:**
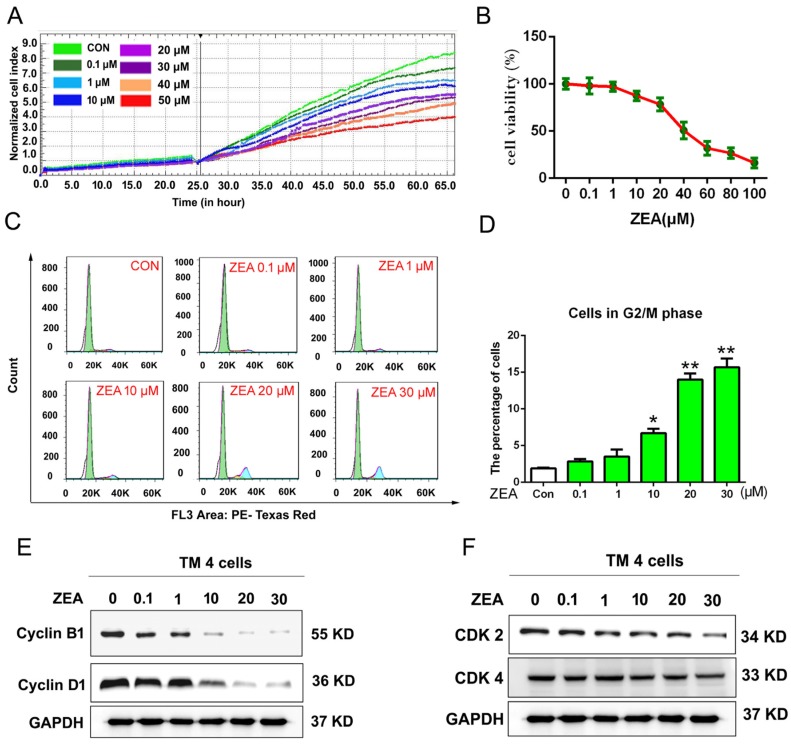
The effect of ZEA on the cell proliferation and cell cyclein TM4 cells. (**A**) Cell proliferation was monitored by the xCELLigence system. (**B**) Cell viability was analyzed by the CCK-8 assay kit. (**C**) The cell cycle distribution was detected by the flow cytometry. (**D**) The analysis of cells on the G2/M phase after cells exposure to the different concentration of ZEA. (**E**,**F**) After treatment with ZEA, cells were harvested to quantitative analyze the expressions of the Cyclin-B1, CyclinD1, CDK2 and CDK4 by using western blotting. Values represent the mean ± S.D. from three different experiments. * *P* < 0.05, ** *P* < 0.01 compared to the control group. Values represent the mean ± S.D. from three different experiments. * *P* < 0.05, ** *P* < 0.01 compared to the control group.

Cell proliferation was monitored by the xCELLigence system in real time and cell counting kit-8 (CCK-8) assay. The data from the xCELLigence system showed (Figure 1A) that after exposure to ZEA, the cell index (CI) value was decreased in a dose-dependent manner compared with the control group. Furthermore, the data from the CCK-8 (Figure 1B)showed that the viability of the TM4 cells was decreased in a dose-dependent manner after treatment with 1, 10, 20, 40, 60, 80 and 100 μM ZEA for 24 h compared with the control group. These data revealed that ZEA could inhibit the growth of TM4 cells. 

To examine the molecular mechanism of ZEA-inhibited cell growth, the distribution of the cell phasewas evaluated by flow cytometric analysis. As shown in Figure 1C,D, ZEA led to a notable accumulation of G2 phase cells in a dose-dependent manner. Additionally, we further detected the effects of ZEA on cell cycle regulatory proteins including Cyclin-B1, Cyclin-D1, CDK2 and CDK4 by western blotting analysis. As shown in Figure 1E,F, after treatment with different concentrations of ZEA for 24 h, the expression of Cyclin-B1, CyclinD1, CDK2 and CDK4 were decreased significantly in a dose-dependent manner. Taken together, ZEA can affect the cell cycle distribution and the expressions of cell cycle regulatory proteins. 

### 2.2. ZEA Can Induce Cell Death and Cell Apoptosis in TM4 Cells 

**Figure 2:**
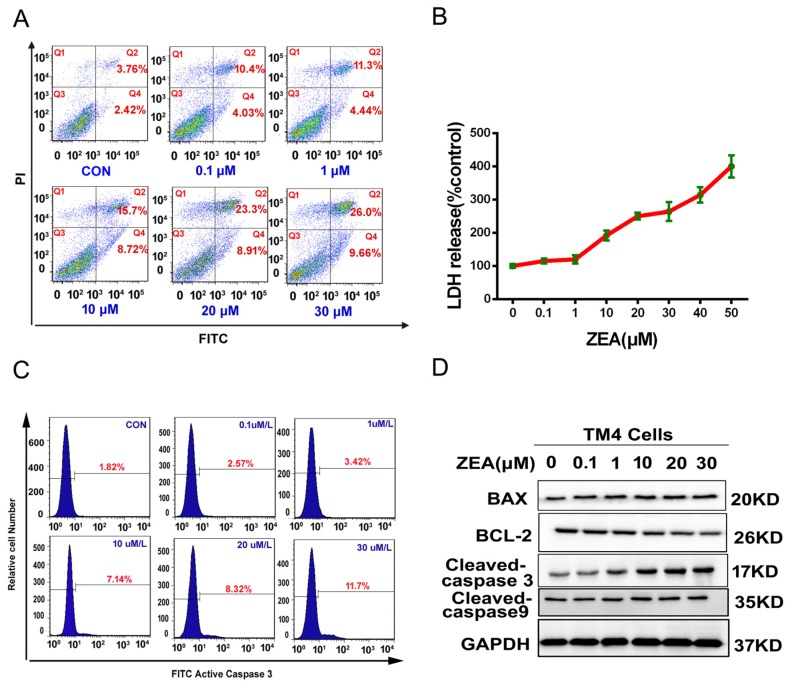
ZEA induced cell death and cell apoptosis. (**A**) The ration of cell death was detected by the LDH release assay kit. (**B**,**D**) ZEA induced apoptosis in TM4 cells. After cell treatment with ZEA for 24 h, cells were harvested to analyze the ratio of apoptosis by using the annexin-V and PI double-staining. (**C**) The activity of caspase-3 was detected by using flow cytometry.

**Figure 3:**
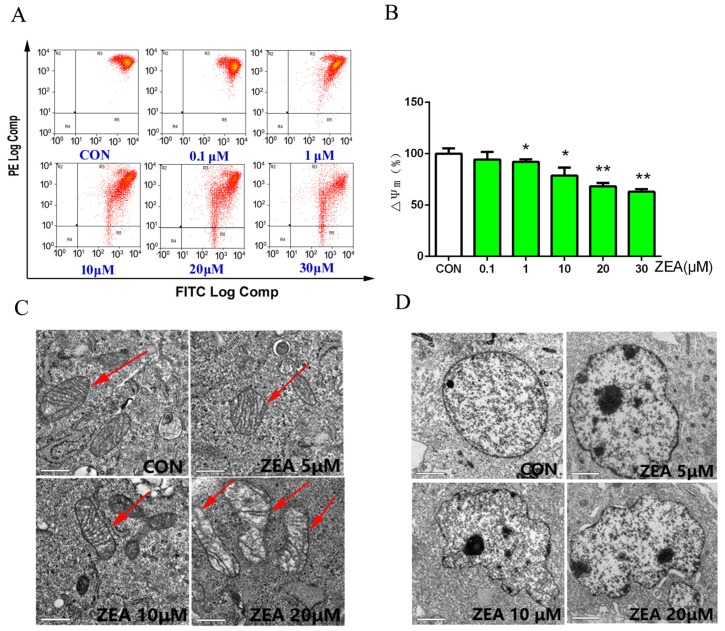
(**A**,**B**) The change of mitochondrial membrane potential was detected by using flow cytometry. (**C**,**D**) The ultra-structural changes were observed by using the electron microscope after the TM4 cells were exposed to ZEA for 24 h. Disruption of mitochondria (red arrows) was observed (×630). Values represent the mean ± S.D. from three different experiments. * *P* < 0.05, ** *P* < 0.01 compared to the control group.

We detected the cell death ratio by using the lactate dehydrogenase (LDH) release assay. As shown in Figure 2B, LDH release increased significantly after treatment with different concentrations of ZEA. In order to detect the mechanism of ZEA causing cell death, the apoptosis parameters were assessed by flow cytometry, western blotting and transmission electron microscopy (TEM). The data from flow cytometry showed that the apoptosis ratio significantly increased from 6.18% in the control group to 35.66% in the 30 μM ZEA-treated group (Figure 2A). Furthermore, the results showed that the activity of caspase-3 was significantly increased (Figure 2C) and the ratio of Bax/Bcl-2, the expressions of cleaved caspase-3 and cleaved caspase-9 were significantly increased in ZEA treatment groups (Figure 2D). The mitochondrial membrane potential significantly decreased in a dose-dependent manner after treatment with different concentrations of ZEA (Figure 3A,B). Furthermore, the results from electron microscopy (Figure 3C,D) showed that for the cells in the control group, the nuclear membranes remained intact and the nuclear chromatin was evenly distributed and the structure of mitochondria and mitochondrial cristae were clearly visible. However, morphologic changes of the cells in the ZEA group were observed, including nuclear fragmentation, chromatin condensation, uneven distribution of nuclear chromatin and aggregation at the periphery of the nucleons. The significant alterations of the mitochondria were the mitochondrial cristae and matrix. The mitochondrial cristae membranes were ruptured and deformed and became blurred and even disappeared. The mitochondrial matrix was also become invisible. These data suggested that ZEA can induce cell death and cell apoptosis.

### 2.3. ZEA-Induced Cell Cycle Arrest and Cell Apoptosis via ROS Generation in TM4 Cells 

**Figure 4:**
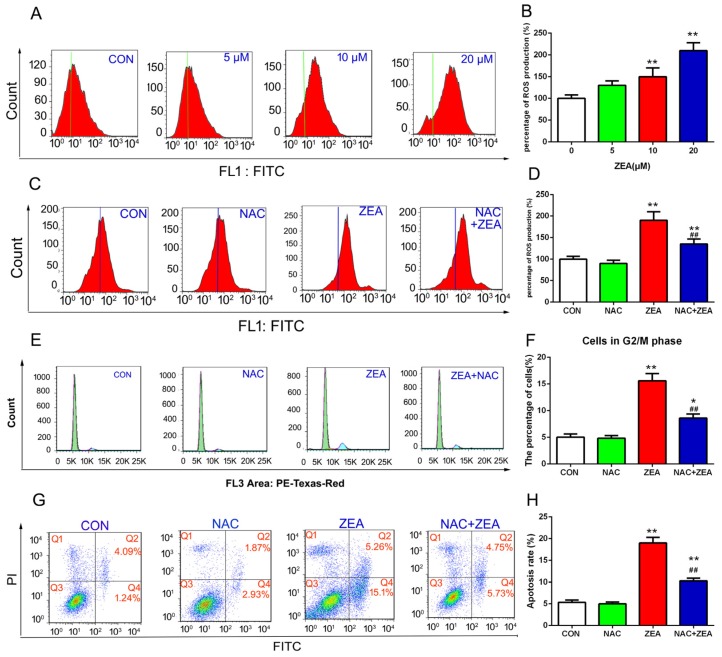
ZEA caused cell cycle arrest and apoptosis via producing ROS. (**A**,**B**) After cell treatment with ZEA, the ROS level was analyzed by flow cytometry. (**C**,**D**) NAC can decrease the level of ROS induced by ZEA. After pre-treatment with 100 μM NAC, the cells were harvested and analyzed at the in intercellular level of ROS flow cytometry. (**E**,**F**) Pre-treatment with NAC can alleviate the accumulation of cells in the G2/M phase. After pre-treatment with 100 μM NAC, the cell cycle was detected by flow cytometry. (**G**,**H**) NAC can decrease the rate of apoptosis. After co-treatment with NAC and ZEA, the cells were harvested and the ration of cell apoptosis was detected using flow cytometry. Values represent the mean ±S.D. from three different experiments. * *P* < 0.05, ** *P* < 0.01 compared to the control group. ## *P* < 0.01 versus the 20 μM ZEA group.

In order to confirm whether the ROS was implicated in the process of ZEA-induced cell cycle arrest and apoptosis, the level of intracellular ROS was analyzed by ROS assay kit. The level of intracellular ROS was increased significantly in a dose-dependent manner after exposing the cells to ZEA (Figure 4A,B). Furthermore, after the pre-treatment with the antioxidant NAC, the intracellular ROS content decreased significantly compared with cells treated with ZEA alone (Figure 4C,D). Then we examined whether increased ROS was involved in the process of ZEA-induced cell cycle arrest and apoptosis. As shown in the Figure 4E,F, the co-treatment with NAC could alleviate the accumulation of cells in the G2/M phase. Similarly, after the co-treatment with NAC, the rate of apoptosis decreased significantly compared with cells treated with ZEA alone (Figure 4G,H). These data suggested that the overproduction of ROS was involved in the process of ZEA-induced cell cycle arrest and cell apoptosis.

### 2.4. ROS-Mediated Cell Cycle Arrest and Apoptosis through ER Stress Pathway in TM4 Cells 

**Figure 5:**
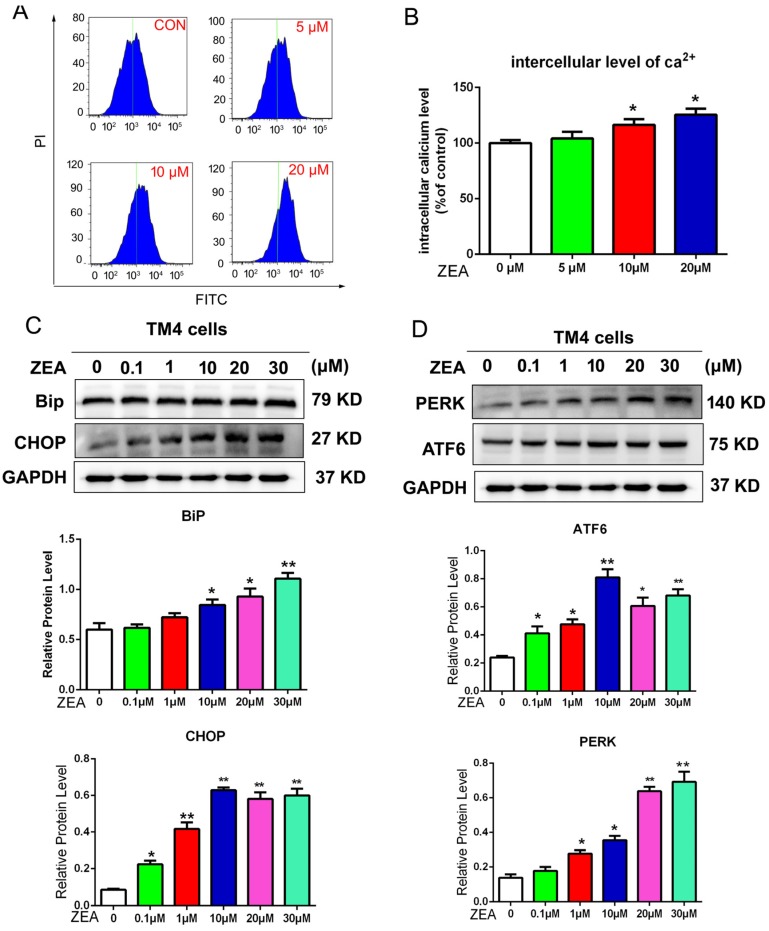
ER stress was stimulated by the ZEA in TM4 cells. (**A**,**B**) After staining using the Fluo-3 AM indicator, the intracellular Ca^2+^ was detected by flow cytometry analysis. (**C**,**D**) The expression of BiP, CHOP, ATF6 and PERK. GAPDH was detected by using western blotting. GAPDH was used as an internal control. Values represent the mean ± S.D. from three different experiments. * *P* < 0.05, ** *P* < 0.01 compared to the control group.

**Figure 6:**
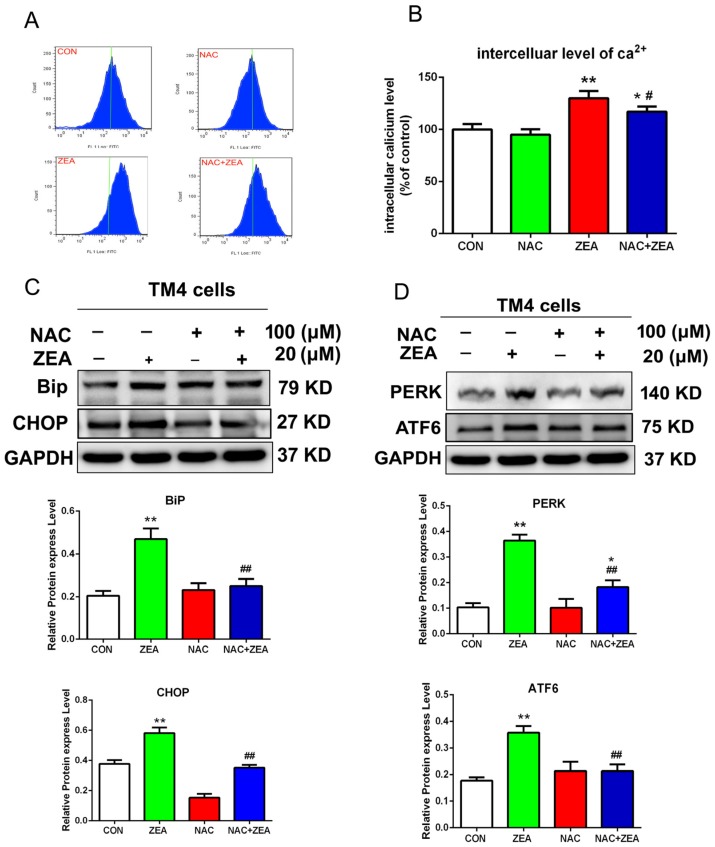
ZEA induced ER stress via ROS. Cells were pre-treated with 100 μM NAC and then exposed to 20 μM ZEA. (**A**,**B**) Pre-treatment with NAC alleviated the increase of intercellular levels of Ca^2+^. (**C**,**D**) NAC remarkably reduced the increase level of BiP, PERK, ATF6 and CHOP. After the cells were treated by the NAC and ZEA, the cells were harvested and the expression of BiP, CHOP, ATF6 and PERK were detected using western blotting. Values represent the mean ± S.D. from three different experiments. * *P* < 0.05, ** *P* < 0.01 compared to the control group; ^#^ *P* < 0.05, ^##^ *P* < 0.01 versus the 20 μM ZEA group.

The ROS/ER stress pathway was revealed as an important pathway for cell proliferation and cell apoptosis. Thus this study determined the effects of ZEA on ER stress. Several molecular indicators of ER stress, including the level of intracellular Ca^2+^ and the expression of BiP, PERK, ATF6 and CHOP were detected using flow cytometry and western blotting. The flow cytometry data demonstrated (Figure 5A,B) that the ZEA-treated cells showed a remarkable increase in intracellular Ca^2+^ compared with the control group. The protein levels of BiP, PERK, ATF6 and CHOP increased significantly in cells treated with ZEA compared with the control group (Figure 5C,D). In order to detect whether the ER stress was induced by ROS, we detected the molecular indicators of ER stress after co-treatment with NAC. The level of intracellular Ca^2+^ also decreased after co-treatment with NAC compared with the ZEA alone treatment group (Figure 6A,B). The results from western blotting suggested (Figure 6C,D) that the protein levels of BiP, PERK, ATF6 and CHOP in the co-treatment (NAC and ZEA) group were remarkably decreased compared with cells exposed to ZEA treatment alone. 

**Figure 7:**
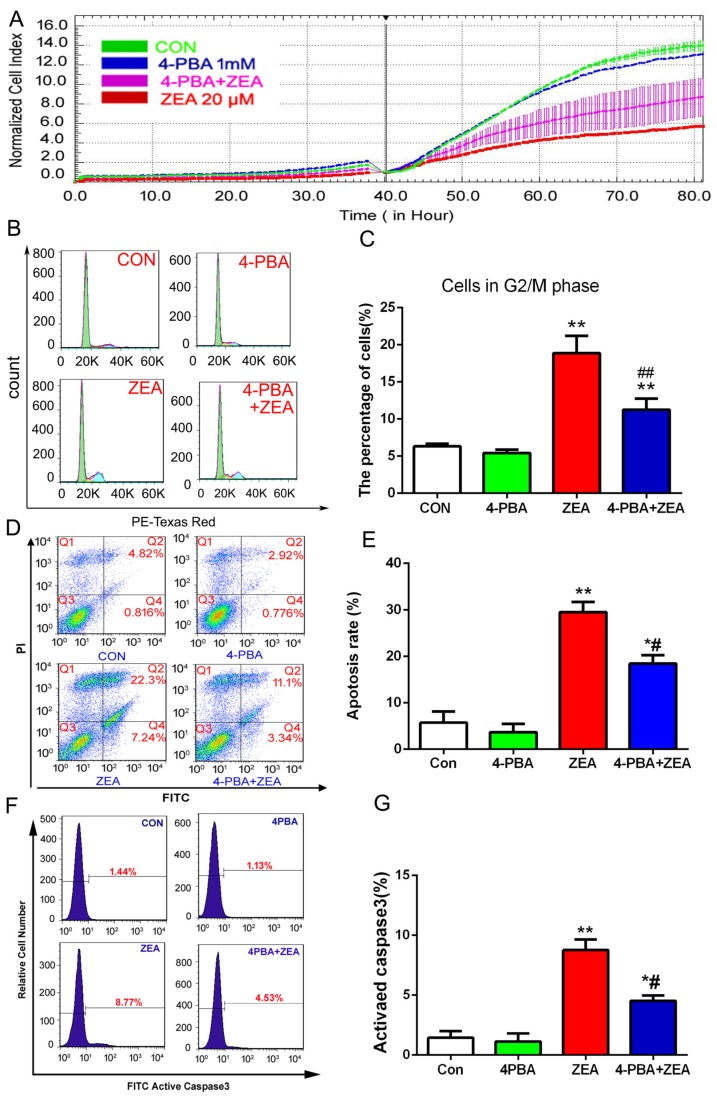
ZEA-induced cell cycle arrest and apoptosis via the ER stress pathway in TM4 cells. (**A**) After pre-treatment with 4-PBA, the cell proliferation was real-time monitored by the xCELLigence system. (**B**) After pre-treatment with 4-PBA, the cell cycle distribution was analyzed by flow cytometry. (**C**) The analysis of cells on the G2/M phase after co-treatment with 4-PBA and ZEA. (**D**,**E**) After pre-treatment with 4-PBA, the cell apoptosis was analyzed by flow cytometry. (**F, G**)After pre-treatment with 4-PBA, the activity of caspase-3 was detected using flow cytometry. Values represent the mean ± S.D. from three different experiments. * *P* < 0.05, ** *P* < 0.01 compared to the control group; ^#^ *P* < 0.05, ^##^ *P* < 0.01 versus the 20 μM ZEA group.

In order to explore the role of ER stress in ZEA-induced TM4 cell cycle arrest and apoptosis, the ER stress inhibitor 4-phenyl butyric acid (4-PBA) was used to alleviated ER stress signals. TM4 cells were pretreated with 1 mM 4-PBA for 1 h and then treated with 20 μM ZEA for an additional 24 h. The data from the xCELLigence system suggested that 4-PBA pre-treatment prevented the cell index decrease induced by ZEA (Figure 7A). The results from flow cytometry demonstrated that 4-PBA pre-treatment reversed the accumulation of cells in the G2/M phase compared with cells treated with ZEA alone (Figure 7B,C).Meanwhile, 4-PBA pre-treatment attenuated TM4 cell apoptosis and active caspase-3 decreased significantly compared with cells treated with ZEA alone (Figure 7D‒G). Taken together, these data suggested that ZEA induced ER stress in TM4 cells through ROS, and ER stress participated in the process of ZEA inhibited the cell proliferation and caused cell cycle arrest and apoptosis.

### 2.5. The ATP/AMPK Pathway Participated in the Process of ZEA-Induced Cell Cycle and Apoptosis 

**Figure 8:**
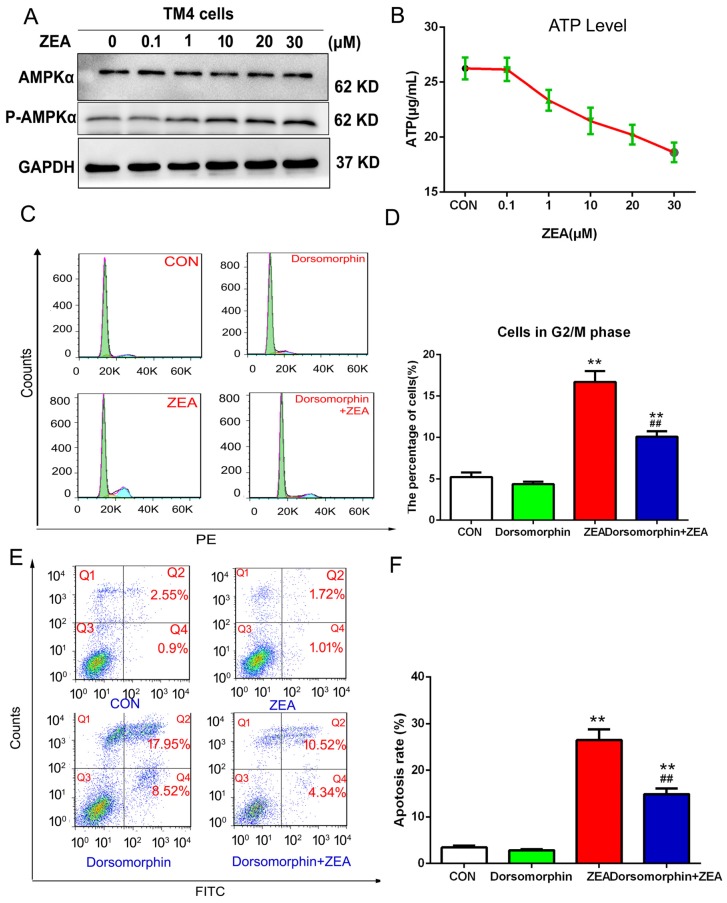
The role of the ATP/AMPK pathway in ZEA-induced cell arrest and apoptosis. (**A**) The effects of ZEA on the expression of AMPK and p-AMPK were detected using western blotting. (**B**) The influence of ZEA on the ATP level was detected using an ATP assay kit. (**C**,**D**) After pre-treatment with the AMPK inhibitor dorsomorphin, the cell cycle distribution was detected using flow cytometry. (**E**,**F**) After pre-treatment with the AMPK inhibitor dorsomorphin, the cell apoptosis was analyzed by flow cytometry. Values represent the mean ± S.D. from three different experiments. * *P* < 0.05, ** *P* < 0.01 compared to the control group; ^#^ *P* < 0.05, ^##^ *P* < 0.01 versus the 20 μM ZEA group.

In order to further investigate the mechanisms of ZEA-induced cell cycle arrest and apoptosis, we detected the effects of ZEA on the ATP/AMAPK signaling pathway. The data from western blotting showed that the ratio of p-AMPK/AMPK increased significantly compared with those of the control group (Figure 8A).The influence of ZEA on the ATP level was detected using an ATP assay kit. As shown in Figure 8B, the intracellular ATP level decreased significantly after treatment with ZEA. To further verify the role of AMPK activation, we blocked AMPK signals by pre-treatment with the AMPK inhibitor dorsomorphin. The number of cells in the G2/M phase decreased significantly in the group treated with the AMPK inhibitor dorsomorphin and ZEA compared with cells treated with ZEA alone (Figure 8C,D). Meanwhile, the rate of apoptosis also decreased significantly after co-treatment with the AMPK inhibitor dorsomorphin compared with cells treated with ZEA alone (Figure 8E,F). Taken together, these data suggest that ZEA could stimulate the ATP/AMPK pathway, which participated in the process of ZEA-induced cell cycle and apoptosis.

### 2.6. The ATP/AMPK Pathway Was Regulated by ER Stress

**Figure 9:**
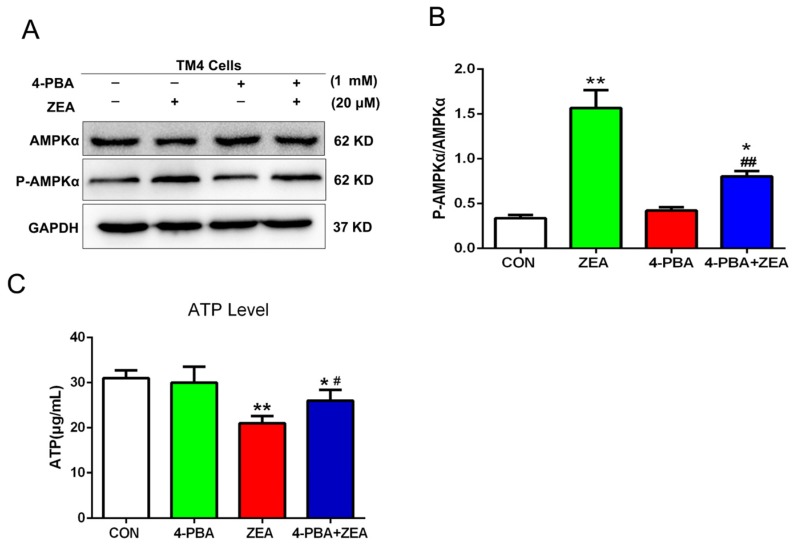
The role of ER stress in the ZEA-stimulated ATP/AMPK pathway. (**A**,**B**) After pre-treatment with 4-PBA, the ratio of P-AMPKα/AMPKα was detected by using western blot. (**C**) After pre-treatment with 4-PBA, the influence of ZEA on the ATP level was detected by using an ATP assay kit. Values represent the mean ± S.D. from three different experiments. * *P* < 0.05, ** *P* < 0.01 compared to the control group; ^#^ *P* < 0.05, ^##^ *P* < 0.01 versus the 20 μM ZEA group.

To clarify the molecular mechanism of ZEA-induced ER stress on the ATP/AMAPK signaling pathway, the relationship between ATP/AMAPK and ER stress was examined by using 4-PBA. As shown in Figure 9A,B after co-treatment with 4-PBA, the ratios of p-AMPK/AMPK decreased significantly compared with the ZEA alone group. Furthermore, the intracellular ATP level increased significantly in the group treated with 4-PBA and ZEA compared with cells treated with ZEA alone (Figure 9C). 

## 3. Discussion

Increasing evidence demonstrates that exposure to ZEA can perturb the differentiation of cells, affect fertility, reduce the generation of reproductive cells and induce the death of germ cells [3,15]. Several recent studies have showed that ZEA can affect cell viability and cause cell death in different cell types [16,17,18]. The detailed molecular and cellular mechanisms of ZEA toxicity have not yet been completely explained. Thus, we aimed to reveal the cytotoxicity of ZEA on TM4 Sertoli cells.

ZEA could exert both proliferative and anti-proliferative activity depending on its different concentrations [19]. Accumulating research has suggested that ZEA can promote cell proliferation and exert a potential tumor-promoting activity in low doses, however a high dose of ZEA can inhibit cell ability and cause cell death, autophagy and apoptosis [9]. Exposure to ZEA at the nano-molar range significantly increased DNA synthesis, the percentage of cells entering proliferative phases and eventually cell proliferation [7]. This study revealed that ZEA could not only inhibit the growth of TM4 cells, but also increase cell death and cell apoptosis significantly. 

Many studies have suggested that the mycotoxin can induce oxidative stress and generate ROS [20,21]. ZEA and its metabolites, including α-ZOL and β-ZOL, caused damage to DNA and produced the generation of ROS [13,21]. In this study, the results suggested that after using NAC to alleviate the oxidative stress, the cell cycle arrest and the cell apoptosis induced by ZEA were reversed. Those data suggested that ZEA induced cell cycle arrest and apoptosis through ROS generation.

Several studies have suggested that ZEA induced cell apoptosis via ER stress and other pathways [11,22]. Many studies have showed [20] that ER stress and caspase-dependent pathways were involved in the process of ZEA causing cell death and apoptosis in HTERT-GLCs and Leydig cells [10,11,22]. Thus, we hypothesized that ZEA induced cell cycle arrest and cell apoptosis via ROS mediated ER stress. The current study suggested that the ER stress was alleviated by using NAC to reverse the generation of ROS. Cell cycle arrest and the rate of apoptosis was decreased after pre-treatment with the ER stress inhibitor 4-PBA. Those results confirmed the hypothesis that the ROS/ER stress path way was involved in this process.

AMPK, mediated by the ration of AMP/ATP, was known as a regulator of cellular energy balance [23]. Recently, it has emerged that the AMPK pathway was involved in the process of cell survival or apoptosis depending on the cell type [23,24,25,26]. Through regulating the carbon source availability, AMPK was involved in the cell cycle process [27]. In the current study, the data suggested that ZEA can activate the ATP/AMPK signal way that was involved in the process of ZEA-induced cell cycle arrest and cell apoptosis. It is known that AMPK was activated by ER stress. A study has showed that ER stress can stimulate the AMPK pathway through Ca^2+^-CaMKKβ [28]. It is tempting to speculate that ZEA-induced AMPK might also be stimulated by ER stress. The data from the current study showed that the AMPK pathway was activated by the ER stress signal. Overall, the results presented here suggest that ER stress might also participate in the cell cycle process and cell apoptosis via activating AMPK under stress conditions.

While the cytotoxicity of ZEA is still not completely explained, the increasing numbers of the study suggested that ZEA and its metabolites can exert estrogen-like effects due to the structural analogy to estrogen, and the strong estrogenic activity was consider to underlie the toxicity of ZEA [29,30]. Furthermore, several studies have showed that since ZEA can cause DNA damage and lesions, which can induce mutations, ZEA also exerts carcinogenic properties. The results presented in the current study provided the evidence that causing oxidative stress and ER stress were important methods for ZEA to exert their cytotoxicity [31,32]. Further explorations are needed to detect how ZEA influences cell growth and death.

## 4. Conclusions

In summary, this study revealed that ZEA induced TM4 cell cycle G2/M arrest and cell apoptosis through ROS- and ER-stress and the ATP/AMPK pathway.

## 5. Materials and Methods 

### 5.1. Reagents Chemicals and Antibodies

Zearalenone (ZEA), and 4-phenylbutyrate (4-PBA), were purchased from Sigma–Aldrich (St. Louis, MO, USA); Fetal bovine serum (FBS) and DMEM/F-12 medium were obtained from Gibco (Grand Island, NY, USA); the cell counting kit-8 (CCK8) was purchased from Dojindo Laboratories (Kumamoto, Japan); the LDH release assay, ROS assay kit (S0033) and ATP assay kit were obtained from Beyotime Institute of Biotechnology (Shanghai, China); and the cell cycle assay caspase 3 activity assay kit and Annexin V/propidium iodide (PI) were purchased from Becton Dickinson Company (BD; Franklin Lakes, NJ, USA). Dorsomorphin dihydrochloride (HY-13418) was purchased from Medchem express (Shanghai, China).

### 5.2. Cell Culture

TM4 cells, a mouse Sertoli cell line obtained from the American Type Culture Collection (ATCC), were maintained in DMEM/F-12 medium supplemented with 10% fetal bovine serum, 1 mM glutamine, 1% penicillin/ streptomycin, and maintained at 37 °C with 5% CO_2_.

### 5.3. Cell Proliferation and Cell Viability 

The cell proliferation was monitored by using the xCELLigence systems and CCK8 assay kit. Briefly, the cells were cultivated in the electric plate at a density of 1 × 104 each well. The cell index of proliferation was detected every 15 min. When the cell were in the logarithm growth stage, the TM4 cells were treated by different concentration of ZEA (0, 1, 10, 20, 40, 60, 80 and 100 μM).The cell proliferation index were normalized at the time when the cells were exposed to ZEA. The CCK8 kit was used to evaluate cell ability. Briefly, the cells were transplanted in the 96 well culture plates at the density of 5 × 104 each well. After the cells were treated by the different concentration of ZEA for 24 h, 10 μL of the CCK-8 solution was added to the cell culture medium at 2 h before the end of treatment. The optical density (OD) of each well was read by the micro-plate absorbance reader at 450 nm.

### 5.4. Cell Cycle Distribution Analysis

After the TM4 cells were treated according to the design of the current study, the cells were harvested and fixed using 70% ethanol. Before staining, the fixed cells were washed twice with PBS and then added the staining solution (PI and RNaseA in PBS) to stain the cells for 30 min at room temperature. The samples were filtered using a 200-mesh nylon filter, and measured using flow cytometry.

### 5.5. Cell Apoptosis Analysis

The ratio of cell apoptosis was analyzed by using the Annexin V-fluorescein isothiocyanate apoptosis detection kit. The cells were digested by trypsin enzyme without Ethylene Diamine Tetraacetic Acid (EDTA) solution and collected into 1.5 mL tubes. The cells were washed by the binding buffer and then resuspended in the binding buffer. The staining solution of Annexin V-FITC and PI were added one by one. The cells were incubated at room temperature for 15 min without light. Using flow cytometry detected the staining cells immediately.

### 5.6. The Level of Intracellular ROS Analysis

The level of ROS was detected by using an ROS assay kit. After treatment, the cells were washed by PBS and resuspened in PBS with 10 μM DCFH. The cells were incubated at 37 °C for 40 min. Then, the cells were washed twice with PBS and resuspended in PBS to detect ROS accumulation using flow cytometry at a wavelength pair of 488/525 nm.

### 5.7. Measurement of Intracellular Ca^2+^

Fluo-3 AM, the fluorescent Ca^2+^ indicator, was used to detect the change of the intracellular Ca^2+^. After the cell was treated by the design of the study, the cells were harvested and washed twice with PBS. The cells were stained with Fluo-3 AM at 37 °C for 30 min without light. Using flow cytometry detected the staining cells immediately.

### 5.8. Western Blotting Analysis

After the cells were treated by the experimental design, the cells were collected using trypsin enzyme-digesting. The whole proteins of the cell were extracted using the RIPA lysis buffer. The concentration of the whole protein was detected and modulated by using the BCA protein assay kit. Collecting the whole protein mixed with loading buffer boiling for 10 min. The extracted proteins were injected into the sodium dodecyl sulfate polyacrylamide gel for separation according to the size of protein and then the proteins were transferred to polyvinylidene fluoride membranes. The membranes were incubated with 5% skimmed milk solution then the membranes were probed with the indicated primary antibodies at 4 °C overnight, washed, and then incubated with secondary antibodies for 2 h at room temperature. The protein signals were detected by ECL detection system. The polyclonal antibodies against GAPDH (2118), CHOP (2895S), PERK (3192s), P-AMPKα (2535S), AMPKα (2532S), Cyclin-B1 (4135), Cyclin-D1 (2978) and CDK2 (9932) were acquired from Cell Signaling Technology (Boston, MA, USA); The polyclonal antibodies against BiP (ab21685), ATF6 (ab203119), CDK4 (ab199728) were purchased from Abcam (Cambridge, MA, USA). 

### 5.9. Transmission Electron Microscopy (TEM)

The cells were fixed using 2.5% glutaraldehyde for 24 h at room temperature. The cells were washed by PBS and then were post-fixed by 1% OsO4 for 20 min. The cells were dehydrated by using a series of ethanol solutions and embedded in the mixture of Epon: alcohol for 2 h. The ultrastructure of the cells was observed by JEM 100CX transmission electron microscope.

#### 5.10. Statistical Analysis

The results were analyzed by using SPSS and presented as the mean ± standard deviation (SD).The difference of different groups were analyzed by using a one-way analysis of variance (ANOVA). *P* < 0.05 was considered statistically significant. 

## Abbreviations

4-PBA	4-phenylbutyrate
α-ZAL	α-Zearalanol
AMPK	AMP-activated protein kinase
ATP	Adenosine triphosphate
CCK-8	cell counting kit-8
CI	Cell index
ER stress	Endoplasmic reticulum stress
FBS	Fetal bovine serum
LDH	lactate dehydrogenase
NAC	*N*-acetyl-L-cysteine
ROS	reactive oxygen species
TEM	Transmission electron microscopy
ZEA	Zearalenone
